# FishFace: interactive atlas of zebrafish craniofacial development at cellular resolution

**DOI:** 10.1186/1471-213X-13-23

**Published:** 2013-05-28

**Authors:** B Frank Eames, April DeLaurier, Bonnie Ullmann, Tyler R Huycke, James T Nichols, John Dowd, Marcie McFadden, Mark M Sasaki, Charles B Kimmel

**Affiliations:** 1 Institute of Neuroscience, University of Oregon, Eugene, OR, USA; 2 Current address: Department of Anatomy & Cell Biology, University of Saskatchewan, Saskatoon, SK, Canada; 3 Current address: Biological and Biomedical Sciences, Harvard University, Cambridge, MA, UK; 4 Current address: Committee on Cancer Biology, University of Chicago, Chicago, IL, USA

**Keywords:** Craniofacial development, Website atlas, Zebrafish, Bone, Cartilage, Skeleton, Evolution

## Abstract

**Background:**

The vertebrate craniofacial skeleton may exhibit anatomical complexity and diversity, but its genesis and evolution can be understood through careful dissection of developmental programs at cellular resolution. Resources are lacking that include introductory overviews of skeletal anatomy coupled with descriptions of craniofacial development at cellular resolution. In addition to providing analytical guidelines for other studies, such an atlas would suggest cellular mechanisms underlying development.

**Description:**

We present the Fish Face Atlas, an online, 3D-interactive atlas of craniofacial development in the zebrafish *Danio rerio*. Alizarin red-stained skulls scanned by fluorescent optical projection tomography and segmented into individual elements provide a resource for understanding the 3D structure of the zebrafish craniofacial skeleton. These data provide the user an anatomical entry point to confocal images of Alizarin red-stained zebrafish with transgenically-labelled pharyngeal arch ectomesenchyme, chondrocytes, and osteoblasts, which illustrate the appearance, morphogenesis, and growth of the mandibular and hyoid cartilages and bones, as viewed in live, anesthetized zebrafish during embryonic and larval development. Confocal image stacks at high magnification during the same stages provide cellular detail and suggest developmental and evolutionary hypotheses.

**Conclusion:**

The FishFace Atlas is a novel learning tool for understanding craniofacial skeletal development, and can serve as a reference for a variety of studies, including comparative and mutational analyses.

## Background

How do elements of the vertebrate craniofacial skeleton arise, grow, and reshape during development, and how do these processes vary during the course of evolution? Answers to these questions are coming from both molecular-genetic and cell-biological approaches, which rely, first of all, on precise description of the developmental events and processes that comprise skeletogenesis. The availability of developmental atlases facilitates such study. Indeed, many descriptive atlases of craniofacial development in various vertebrate species have been published [1-4]. In today’s world, internet atlases are extremely useful, in part because they are publicly accessible around the world, but also because they can be formatted so that a user interacts with the data in unique ways, such as with freely-rotatable 3D models (e.g., Mouse Limb Atlas: http://www.nimr.mrc.ac.uk/3dlimb/; [5]).

Zebrafish, with patterns of development conserved among all vertebrates, with favorable attributes for phenotypic analyses of development, and with a sophisticated knowledge by the research community of its genetics and genomics, provides a powerful animal model for learning about craniofacial development. Online zebrafish atlases include the Zebrafish Atlas (zfatlas.psu.edu); 3D Atlas of Zebrafish Vasculature Anatomy (http://uvo.nichd.nih.gov/atlas.html); the Zebrafish Brain Atlas (http://www.ucl.ac.uk/zebrafish-group/zebrafishbrain/index.php); the Atlas of Zebrafish Anatomy (http://www.zebrafish.uni-freiburg.de/anatomy.html); the Atlas of Zebrafish Development (http://bio-imaging.liacs.nl/liacsatlas.html); the Zebrafish Anatomy Portal (http://www.zfap.org); and the FishNet 3D developmental atlas (http://www.fishnet.org.au/index.shtml). None of these atlases, however, currently focus on development of the craniofacial skeleton.

Zebrafish craniofacial development can be detailed with an investigative method unsurpassed in accuracy and sensitivity. Using current transgenic approaches in combination with vital dye staining, one can examine craniofacial skeletal elements in exquisite cellular detail during an extended period of development within living, intact zebrafish embryos and larvae. Here, we describe a high-resolution developmental atlas of the zebrafish craniofacial skeleton, the FishFace Atlas (https://www.facebase.org/fishface/home), to serve as a guide for anatomical and cellular studies of craniofacial development.

## Utility

### Summary of FishFace Atlas website organization

The FishFace Atlas focusses on cellular level anatomy of the developing skeleton in the first two pharyngeal arches. It uses confocal imaging of living specimens. To assist the user unfamiliar with fish craniofacial anatomy, FishFace also provides an overview of the structure of the skull, featuring optical projection tomography (OPT) of preserved material. In this “Skull Anatomy” section, fluorescent OPT images of Alizarin red-stained samples show skull bones of middle and late larval, and adult, zebrafish (https://www.facebase.org/fishface/opt_data; for clarification of the relationship between the FishFace Atlas and FaceBase hub, please see (Additional file 1)). Furthermore, a model of larval craniofacial bones was created by segmentation of 3D reconstructed OPT data, and this model can be freely rotated and virtually dissected in a user-defined fashion (https://www.facebase.org/fishface/Viewer).

The “Arch 1 and 2 Development” section of FishFace complements data from “Skull Anatomy”, including ‘overview’ series of low magnification confocal image stacks through the two most anterior pharyngeal arches and through the craniofacial skeleton derived from these arches. These images show transgenically-labelled cells in living embryos and larvae, and all ontological terms in their descriptions link directly to standardized definitions on the Zebrafish Information Network (http://zfin.org/). One such ‘overview’ is a series of embryonic zebrafish at preskeletal stages showing transgenically-labelled neural crest-derived mesenchymal cells that eventually give rise to craniofacial skeletal elements (https://www.facebase.org/fishface/early_arches). The second ‘overview’ series illustrates developing skeletal elements using transgenically-labelled chondrocytes and Alizarin-red stained bone matrix (https://www.facebase.org/fishface/arch1_and_2).

The heart of the FishFace Atlas is the “Element Development” section, which uses high magnification confocal image stacks of transgenically-labelled chondrocytes or osteoblasts, along with Alizarin red-stained mineralized bone matrix, to offer insight into the genesis of the anatomical complexity demonstrated by the OPT and low magnification ‘overview’ confocal data. This ‘element development’ section adds cellular detail to selected skeletal elements in pharyngeal arch one and pharyngeal arch two, tracking ontogenetic sequences of the following individual cartilages and bones as they develop during embryonic and larval stages:

palatoquadrate (https://www.facebase.org/fishface/palatoquadrate);

Meckel’s cartilage (https://www.facebase.org/fishface/meckel);

hyosymplectic (https://www.facebase.org/fishface/hyosymplectic);

ceratohyal (https://www.facebase.org/fishface/ceratohyal); and

opercle (https://www.facebase.org/fishface/op_and_bsr_series).

By studying elements in cellular detail, a much more precise description can be made of the dramatic changes to element growth and shaping that occur in early developmental stages. Most images in FishFace have links to movies playing slice by slice through each confocal stack of the z-series that was used to generate that particular confocal projection. Hence, the FishFace Atlas provides the community with a valuable, interactive resource with which the user can understand not only the complex 3D anatomical relationships of skeletal elements, but also the underlying cellular organization, in the developing zebrafish craniofacial skeleton.

### Skull Anatomy: an interactive model of zebrafish craniofacial skeletal anatomy

**Figure 1:**
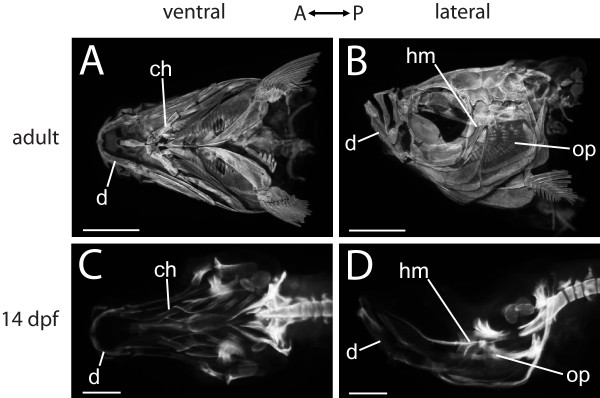
**OPT data provide an overview of zebrafish craniofacial skeletal anatomy.** Ventral (**A**, **C**) and lateral (**B**, **D**) views of zebrafish heads from OPT data demonstrate the decrease in complexity of skeletal elements when earlier specimens are compared with older specimens. While the adult head is covered by bones (**A**, **B**), only a small number of ossifications are visible in the ventral region of the 14 dpf zebrafish craniofacial skeleton (**C**, **D**). Representative skeletal elements are indicated. Abbreviations: A = anterior; ch = ceratohyal; d = dentary; dpf = days post-fertilization; hm = hyomandibula; op = opercle; P = posterior. Scale bars: **A**,**B** = 1 mm; **C**,**D** = 200 μm.

To provide an understanding of the overall anatomy of the zebrafish craniofacial skeleton, we used fluorescent optical projection tomography (OPT) imaging of Alizarin red-stained adult, 18 days post-fertilization (dpf), and 14 dpf zebrafish heads. The adult zebrafish head has a beautifully complex assortment of 74 bones covering almost the entire dorsal, lateral, and ventral surfaces (Figure 1A, B; [6]). A general theme of the FishFace Atlas is to facilitate understanding of such complexity by tracking structures earlier in development, when the anatomy is simpler. By 14 dpf, for example, OPT imaging demonstrates that the craniofacial skeleton of the zebrafish consists principally of a small number of lateral and ventral bones in the pharyngeal arches (Figure 1C, D). To reveal the complex relationships of the many bones of the zebrafish craniofacial skeleton, the user may view movies that cycle through the raw data of the initial scans, which take an image from each of 400 rotational angles of the specimen (e.g., links on https://www.facebase.org/fishface/opt_data). Downloadable movies on this webpage (please see “OPT imaging and processing” within ‘Construction and content’) also enable the user to progress from anterior to posterior through 3D reconstructions of the OPT data.

**Figure 2:**
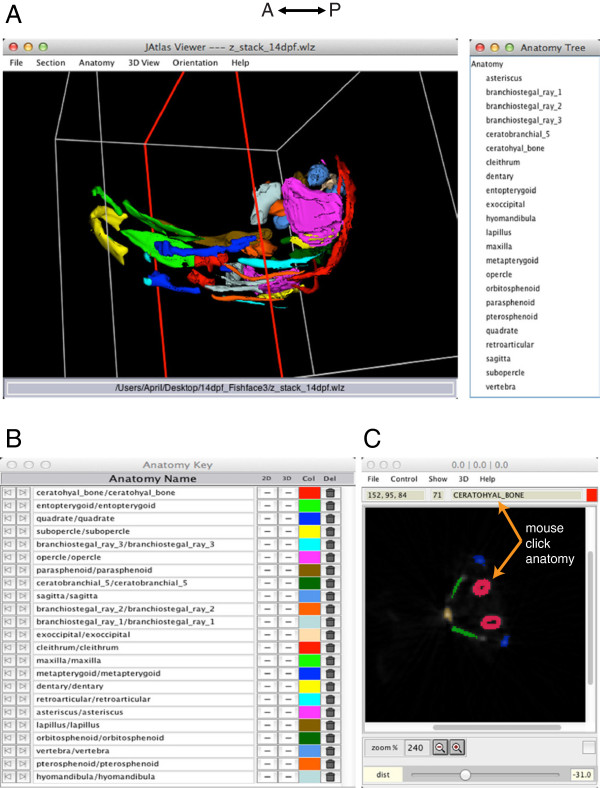
**Skeletal anatomy of the zebrafish head at 14 dpf visualized using Jatlas Viewer.** The interactive 3D model of skeletal structures (**A**) can be rotated in a user-defined fashion with a click and drag of the mouse. The Anatomy Tree (right) lists all of the structures displayed in the 3D view. The Anatomy Key (**B**) shows the name of each structure in the 3D model and its color code. Structures can be switched on and off in the 2D and 3D viewer windows by clicking on "+/-" in the 2D and 3D columns of the Anatomy Key. Structures can be removed and replaced from the 3D and 2D viewers by clicking on the icon in the Del column. Colors can be changed by clicking in the Col column, which opens a palette of colors to choose from. In the 2D section view (**C**), structures shown in the 3D viewer are displayed in the same colors. With the 'mouse-click anatomy' function, users can use their mouse to click structures on the 2D section, and the name appears at the top of the 2D viewer window. The plane of section is shown as a red outline in the 3D model (**A**), and can be changed by adjusting 'yaw' and 'pitch'.

Finally, we created from the 14 dpf 3D reconstructions a model of zebrafish skeletal anatomy, which the user can visualize through the JAtlas Viewer program (https://www.facebase.org/fishface/Viewer). In this highly-interactive format, the user can build, rotate, magnify, and virtually dissect elements of the juvenile zebrafish craniofacial skeleton (Figure 2A). Individual bones are color-coded and annotated, and users manipulate 3D models and generate virtual 2D sections through the specimen (Figure 2B, C). Using the 'mouse-click anatomy' in the section viewer (under Show/mouse-click anatomy), users can use their mouse to click on a feature in the 2D section view, and the name of the element appears at the top of the viewer window. We anticipate that 2D views will be helpful especially for users seeking annotation tools while interpreting histological data.

### Arch 1 and 2 Development: arch ectomesenchyme to cartilages and bones

To understand development of the first two pharyngeal arches, mandibular and hyoid, the FishFace Atlas uses confocal imaging in living preparations. Generated from transgenic zebrafish, the images illustrate cellular details not only of the developing pharyngeal arches, but also of the cartilages and bones that form within them. As such, the confocal data provide a nice complement to the OPT data, which visualizes mineralized matrix of the developing zebrafish craniofacial skeleton. Two sets of low-magnification confocal images help the user transition from the OPT dataset and show how craniofacial morphology changes with time. One covers six stages before, and the other eight stages after, skeletal elements become individually recognizable. Both generally include ventral and lateral views.

**Figure 3:**
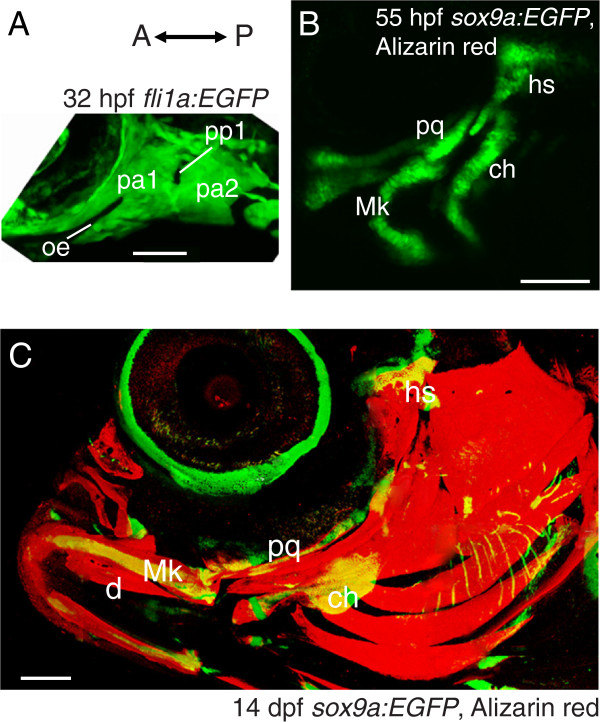
**Low magnification confocal images demonstrate dynamics of morphology and complexity during zebrafish craniofacial development.** Lateral view of *fli1a*:*EGFP* zebrafish at 32 hpf (**A**) illustrates apparent simple and homogenous arrangement of cells in the anterior two pharyngeal arches. Lateral view of Alizarin red-stained *sox9a*:*EGFP* zebrafish at 55 hpf (**B**) demonstrates that most cartilages, including dorsal and ventral elements, have begun to form in these pharyngeal arches, while no bones are visible. Compared to 55 hpf, Alizarin red-stained *sox9a*:*EGFP* zebrafish by 14 dpf (**C**) show that cartilage elements have changed in morphology and size, but the addition of many bones results in a major increase in complexity. Abbreviations: A= anterior; ch = ceratohyal; d = dentary; dpf = days post-fertilization; hpf = hours post-fertilization; hs = hyosymplectic; Mk = Meckel’s cartilage; oe = oral ectoderm; P = posterior; pa = pharyngeal arch; pp = pharyngeal pouch; pq = palatoquadrate. Scale bars: **A** = 50 μm; **B** = 100 μm; **C** = 200 μm.

Selected lateral views from FishFace show how zebrafish craniofacial morphology undergoes remarkable changes during embryonic and larval periods (Figure 3). Embryonic pharyngeal arches (Figure 3A), made largely from neural crest-derived ectomesenchyme expressing *fli1a*:*EGFP*, appear relatively homogeneous and simply organized at 32 hpf (even though developmental patterning studies show that they are anything but homogeneous and simple at this stage [7]). About a day later (Figure 3B, 55 hpf), expression of *sox9a*:*EGFP* reveals early cartilage rudiments developing in these arches. Two cartilages, one dorsal and one ventral, have formed in each arch on each side of the body. No bones are yet mineralized. FishFace then illustrates how this early skeletal pattern progressively elaborates and increases in complexity, such that by about midway through larval development (Figure 3C, 14 dpf) the morphology looks completely different from earlier stages.

### Element Development: detailed single-cell resolution of imaging suggests developmental and evolutionary hypotheses of individual skeletal elements

The final component of the FishFace Atlas, and the most novel, is a set of ‘element’ pages. This resource includes abundant images at high magnification that provide cellular resolution to accompany the lower magnification ‘overview’ images just discussed. By using the ‘overview’ and ‘element’ parts of the atlas together, the viewer is able to directly connect developing element morphologies with changes in cellular patterning, such as cellular arrangements, shapes, and sizes. Together, these help to elucidate the cellular basis of morphogenesis and growth. Here we use the mandible, the lower jaw, to show that imaging with cellular resolution allows one to support hypotheses that then can be tested by more directed and analytically based study. The mandible represents an interesting case study, due to its complex developmental and evolutionary history [1,8-10].

**Figure 4:**
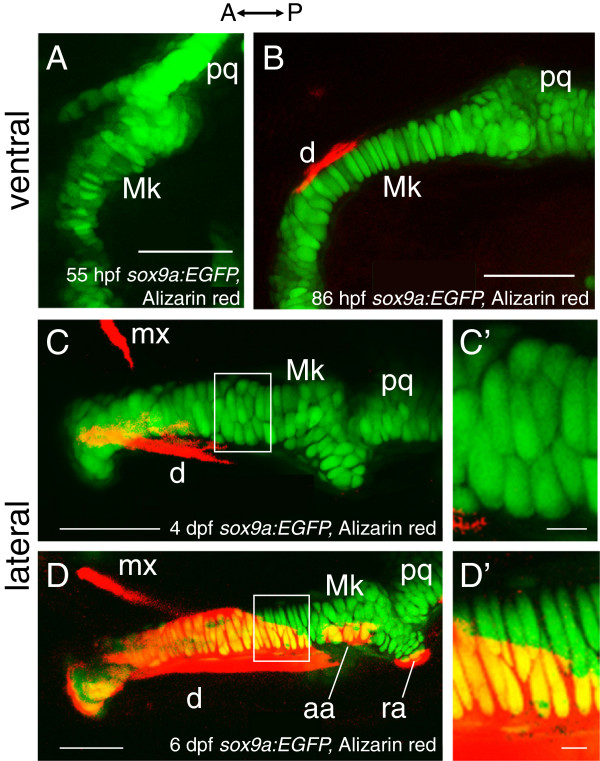
**Chondrocyte intercalation and proliferation seem to drive cartilage growth in late embryonic and early larval stages.** Confocal images in ventral view of Alizarin red-stained *sox9a*:*EGFP* mandibles suggest that early phases of Meckel’s cartilage morphogenesis may be achieved by chondrocyte intercalation. By 55 hpf (**A**), chondrocytes in Meckel’s cartilage do not appear in ordered rows, as generally two to three cells span the mediolateral width of the element. By 86 hpf (**B**), Meckel’s cartilage has grown in length and is relatively thinner from that seen at 55 hpf, and many of its chondrocytes now span the mediolateral width in a cellular stack. Confocal images in lateral view of Alizarin red-stained *sox9a*:*EGFP* mandibles suggest that later phases of Meckel’s cartilage morphogenesis may be achieved by chondrocyte proliferation. Chondrocytes of Meckel’s cartilage are separated clearly from each other by 4 dpf (**C**). By 6 dpf, proliferation of chondrocytes about mid-way along the anterior-posterior length of Meckel’s cartilage (**D**) is suggested by the presence of chondrocyte doublets. These doublets are separated from each other by a layer of presumed extracellular matrix that appears much thinner than that observed between the doublets. **C**’ and **D**’ show high magnification views of the boxed regions in **C** and **D**, respectively. Abbreviations: A = anterior; aa = anguloarticular; d = dentary; dpf = days post-fertilization; hpf = hours post-fertilization; Mk = Meckel’s cartilage; mx = maxilla; P = posterior; pq = palatoquadrate; ra = retroarticular. Scale bars: **A**-**D** = 50 μm; **C**’,**D**’ = 5 μm.

The first mandibular skeletal element is Meckel’s cartilage, present by 55 hpf (Figure 4A). During the following days, the cartilage grows in size and reshapes, becoming longer and relatively thinner (Figure 4, compare A and B; and view the many more images showing Meckel’s cartilage in the Fishface Atlas under “Element Development”). Cells might mediate this kind of shape change, known as convergence and extension, by one or more of several distinct activities; they might reshape, migrate, and/or intercalate with one another. Detailed analyses show that cellular intercalation is the predominant cellular basis of notochord convergence and extension [11,12], and the FishFace images motivate intercalation as a leading hypothesis to explain convergence and extension of Meckel’s cartilage as well. At early stages (Figure 4A), the chondrocytes are in a seemingly disordered arrangement, generally more than two of them spanning the mediolateral thickness of Meckel’s cartilage. Within two days (Figure 4B), a longer, thinner cellular array has become more ordered, with long stretches of the cartilage comprised of a one-cell wide stack.

Cellular imaging also provides support for hypotheses explaining growth in size of individual cartilages. As is well known, chondrocytes greatly enlarge in size, especially during early stages of differentiation morphogenesis, and the number of chondrocytes in growing cartilages increases as well (e.g., as quantified for the zebrafish symplectic cartilage [4]). New chondrocytes can come from the perichondrium (resulting in appositional growth), and they can come from cell divisions of the chondrocytes themselves (resulting in interstitial growth). Interstitial growth yields distinctive chondrocyte doublets, which at first occupy single lacunae within the matrix. FishFace images support interstitial growth of Meckel’s cartilage (but do not exclude appositional growth). Compared to arrangements of chondrocytes and their separating matrix in Meckel’s cartilage at 4 dpf (Figure 4C,C’), images at 6 dpf capture what seems to be a nest of doublets (Figure 4D,D’; essentially all of the cells in Figure 4D’ are included in this nest), and suggest that cell-cycles are locally synchronous.

**Figure 5:**
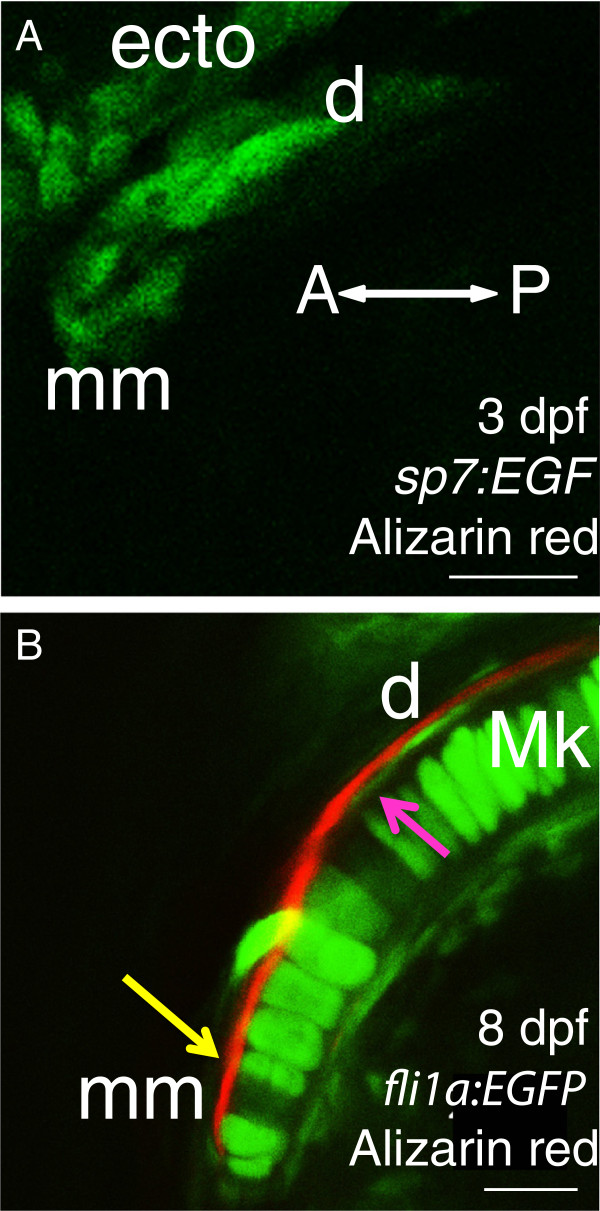
**Cellular resolution of bones in the anterior Meckel**’**s cartilage suggests that the dentary has fused with the mentomeckelian bone from the earliest stages of osteogenesis.** Before bone matrix is detected by Alizarin red, only one group of osteoblasts is apparent adjacent to Meckel’s cartilage in 3 dpf Alizarin red-stained *sp7*:*EGFP* larvae from a ventral view (**A**). In a confocal slice of the anterior region of Meckel’s cartilage in 8 dpf Alizarin red-stained *fli1a*: *EGFP* larvae (**B**), a single bone is visible from a ventral view, but its association with chondrocytes of Meckel’s cartilage appears to vary anteroposteriorly. Anteriorly, bone lies immediately adjacent to chondrocytes (yellow arrow), while more posteriorly, the bone appears to be separated from chondrocytes by cells of the presumptive perichondrium (magenta arrow). Abbreviations: A = anterior; d = dentary; dpf = days post-fertilization; ecto = ectoderm; Mk = Meckel’s cartilage; mm = mentomeckelian; P = posterior. Scale bars: **A**,**B** = 20 μm.

Besides developmental hypotheses, such as the two just proposed for cartilage morphogenesis and growth, the precision afforded by live confocal imaging at cellular resolution can motivate and support evolutionary hypotheses. For example, Jollie regards as a teleost synapomorphy that the most prominent bone in the mandible, the dermal dentary, is actually compound, whereby its anterior end is comprised of a chondral element, the mentomeckelian [13]. As evidence, some species (e.g., *Esox*, pike; *Salmo*, salmon) have a separate mentomeckelian in larvae, which then becomes fused with the dentary [14]. However, imaging in the FishFace Atlas suggests a different mechanism in zebrafish, leading to the hypothesis that a single compound (i.e., fused) element is present from the earliest stages of bone formation. At 3 dpf, before any matrix is detected by the sensitive vital Alizarin red staining, only one local cluster, not two, of early osteoblasts is apparent using the *sp7*:*EGFP* transgenic zebrafish (Figure 5A). After mineralization (4 dpf and later), only a single Alizarin red-labelled bone is detected (Figure 5B), but labelling of skeletogenic mesenchyme with *fli1a*:*EGFP* zebrafish demonstrate that two regions of the bone have different associations with the underlying chondrocytes. Anteriorly, the bone lies directly adjacent to the chondrocytes in the manner of a chondral bone (the mentomeckelian; yellow arrow, Figure 5B). More posteriorly, the bone appears to pass through the perichondrium to lie superficial to it, with transgenic cells visible between the bone and the chondrocytes, in the manner of a dermal bone (the dentary; majenta arrow, Figure 5B). If supported by definitive analyses, this zebrafish condition of the mentomeckelian fusing with the dentary from its earliest stages would provide new evidence of how bones might be ‘lost’ during evolution, widespread among vertebrates, including humans (see Discussion).

We emphasize that the Fishface images by themselves motivate, but do not establish, the validity of the above hypotheses. All three hypotheses we propose here are amenable to rigorous testing. For example, in time-lapse recordings of appropriate strains of transgenic zebrafish, one can observe directly, in the intact fish, cell intercalations mediating convergence and extension [11], waves of cell division [15], and whether osteoblasts are initially recruited to two adjacent bone primordia that quickly coalesce, or to a single one [16].

## Discussion

The FishFace Atlas is an interactive, 3D tool for understanding aspects of the complex anatomy of the developing zebrafish craniofacial skeleton at cellular resolution. FishFace builds on and elucidates the foundation set out primarily in Cubbage and Mabee (1996), who comprehensively described skeletal elements in the zebrafish skull from fixed preparations stained for cartilage and bone through adult stages, but also in other studies that included cartilage development [17] and more detailed investigations of both cartilage [4] and bone [16,18] morphogenesis and growth. FishFace first provides overviews of the whole skull and then focusses on the first and second pharyngeal arches, the region that probably has been the most thoroughly examined region of the zebrafish skull, and which promises to continue to yield fundamental information on its genetics, patterning, morphogenesis and growth [7]. Therefore, the FishFace Atlas should be particularly useful in comparative and mutational analyses where there is interest in understanding not only the genetic basis, but also the cellular basis of skeletogenesis and patterning.

The imaging resolution of skeletal elements in the FishFace Atlas illuminates not only cellular behaviors that drive early craniofacial morphogenesis in living embryos and larvae, but also potential evolutionary mechanisms. Previous studies of craniofacial development have shown that cell migration, aggregation, intercalation, proliferation, enlargement, recruitment, and death, all play crucial roles in generating proper morphology [4,16,19-22]. As we point out for the mandible, the FishFace images support developmental hypotheses explaining reshaping and organized growth of its cartilaginous element, Meckel’s cartilage. Cellular intercalations may drive convergence and extension, and interstitial growth, perhaps including mitotic waves, might contribute to growth. The images also support a compound nature of the dentary, a posterior dermal element fused with an anterior chondral one. We hypothesize that the fusion is present in the osteoblast primordium (i.e., condensation) that initially makes the bone, which is consistent with a mechanism of skeletal evolution expounded by Atchley and Hall [23]. Furthermore, Patterson, in his important review of skeletal element evolution, proposes that often bone ‘loss’ is actually fusion and may occur in evolutionary sequences [24]. The primitive condition is multiple bones: two in the case of anterior mandibular bones, with independent mentomeckelian and dentary bones apparent in teleost outgroups, such as the bowfin *Amia*. Then a step occurs that Patterson calls “ontogenetic fusion”, in which separate bone rudiments are present in early ontogeny and then fuse (as for the trout and pike; [14]). Finally, as a yet more derived condition, there is “phylogenetic fusion” in Patterson’s words; where the ancestor had two (or more) bones, the descendant at all developmental stages has but a single one. Patterson notes that convincing evidence for phylogenetic fusion is lacking [24]. As pointed out above, time-lapse studies in zebrafish could provide this evidence, an analysis motivated by imaging in the FishFace Atlas.

## Conclusions

In order to increase understanding of the development of the zebrafish craniofacial skeleton at cellular resolution, the FishFace Atlas is a website that presents images generated by confocal microscopy and optical projection tomography (OPT) in a user-interactive format. We anticipate its utility as a reference for mutational and evolutionary analyses.

## Construction and Content

### Larval rearing and quality control

All zebrafish lines were maintained, and zebrafish embryos and larvae were raised, according to established protocols [4,25] with IACUC approval (see http://zfin.org/zf_info/zfbook/chapt3/3.2.html). Briefly, up to 20 larvae in 150 ml EM were fed paramecia in 250 ml beakers from 4 days post-fertilization (dpf) to 9 dpf, when they were shifted to mouse cages and fed paramecia and baby brine shrimp until 15 dpf, when they were fed only brine shrimp. Regarding quality control of larval rearing, co-authors followed their strict published methods [18] to monitor general hallmarks of larval health (e.g., swim bladder, prey capture) in our nursery, and standard length measurements were taken for each imaged specimen. Regarding quality control of images included in FishFace, co-authors of the paper collected the images, which were selected for Atlas inclusion by consensus among co-authors, based upon 1) how well the images reflected average development of multiple specimens, often from multiple independent clutches; and 2) how consistent the data were with relevant published literature (e.g., [4,6,17]).

### Alizarin red staining

For OPT imaging of mineralized matrix, fixed zebrafish specimens were stained as described previously [26]. Tissues were fixed overnight in 4% PFA, washed for an hour in 1% KOH, bleached in 3% H_2_O_2_/0.5% KOH for 40 min. with lids open, washed in 1% KOH, stained overnight in 0.003% Alizarin red in 1% KOH, and de-stained in 1% KOH. After eyes were removed, heads were rinsed in water, embedded in low melting point agarose (Invitrogen), washed twice in methanol, and cleared in benzyl alcohol:benzyl benzoate (1:2). For confocal imaging of mineralized matrix in live specimens, zebrafish larvae were maintained in Embryo Medium supplemented with 0.005% Alizarin red and 0.01 M HEPES at least two hours, and often overnight, prior to confocal imaging [18].

### OPT imaging and processing

Images of craniofacial bones of 14 dpf, 18 dpf, and adult zebrafish were captured using Bioptonics OPT Scanner 3001 M (MRC Technology). 3D reconstructions of raw data were made using NRecon (MRC Technology). Segmentations of skeletal elements in reconstructions were made using Amira 5.2.2 (Visage Imaging), and movies highlighting specific skeletal elements were created in Amira and QuickTime Player. Movies showing progression through virtual sections of the reconstruction were created in QuickTime Player (Apple). For information on how to download movies from FishFace for importation into ImageJ, please see the Additional file 1.

### Transgenic animals

All transgenic fish were maintained on the AB background.

The transgenic zebrafish line *sox9a*^*zc81Tg*^, which we refer to here as *sox9a*:*EGFP*, was created using Gateway technology and includes a portion of the *foxp2* promoter driving EGFP expression (*Tg*(*foxp2*.*A*:*EGFP*); [27]). Please see Construction and content and Additional file 2 for details on how *sox9a*:*EGFP* was isolated from the transgenic line *Tg*(*foxp2*.*A*:*EGFP*)*zc42*[27].

The transgenic zebrafish line *Tg*(*sp7*:*EGFP*)*b1212*, which we refer to here as *sp7*:*EGFP*, was created using BAC transgenesis and includes a large portion of the *sp7* promoter driving EGFP expression [28].

The transgenic zebrafish line *Tg*(*fli1a*:*EGFP*)*y1*, which we refer to here as *fli1a*:*EGFP*, was created by micro-injecting a construct including a portion of the *fli1a* promoter driving EGFP expression [29] and has been used widely as a marker of neural crest cells and their derivatives, as well as of endothelial cells (e.g., [19,30,31]).

### Cloning genomic locus of sox9a^zc81Tg^

Genomic DNA was isolated from fins of three adult *sox9a*^*zc81Tg*^, which we refer to here as *sox9a*:*EGFP*, zebrafish with cartilage expression and of one non-glowing sibling adult zebrafish (QIAGEN DNeasy, QIAGEN Inc.). 1 μg gDNA from each of these four samples was digested with *AluI* or *HaeIII* in 40 μl at 37C for 3 hours. 0.5 μg of digested gDNA was ligated with 4 μl T4 DNA ligase (Epicentre) in 500 μl overnight at 16 C. The reaction was concentrated using Zymo-5 column (Zymo Research Corp.) and resuspended in 20 μl for use as PCR template. Inverse PCR was carried out according to published protocols for cloning *Tol2* insertion sites into the zebrafish genome [32,33]. First-round PCR primers were either [Tol2-3’invf2 + Tol2-3’/r1] or [Tol2-3’invf1 + Tol2-3’invr1], while second-round PCR primers were either [Tol2-3’/f2 + Tol2-3’invr2] or [Tol2-3’invf2 + Tol2-3’invr2] [32,33]. No PCR products were obtained from non-transgenic fish. Many clones from products of the different PCR primer pairs were sequenced from each transgenic fish, and sequences BLASTed to the same location (Chr12: 2278311; Zv9, http://www.ensembl.org).

### Confocal microscopy

Imaging of live zebrafish specimens at various time points on 1, 2, 3, 4, 6, 8, 10, 14, and 21 dpf was conducted using either a Zeiss LSM 5 Pascal confocal or Leica SD6000 spinning disk confocal with Borealis illumination technology. In order to visualize Alizarin red staining with most sensitivity, pinhole and/or detector gain settings were adjusted by hand to levels just below those that showed red fluorescence in surrounding, non-mineralized tissues. Maximum projections were made from stacks of images that demonstrated the entire depth of the element(s) under focus. Movies showing progression through all images of the stack were created in Pascal (Carl Zeiss, Inc.) or Metamorph (Leica Microsystems) and sometimes were edited in ImageJ (NIH) for orientation and cropping.

### Comments on developmental generalizations

The FishFace Atlas illustrates a generalized developmental sequence of the appearance of specific skeletal elements, but there are caveats to such generalizations. First, development is a dynamic, variable process. Apart from time-lapse movies, temporal series of images are merely snapshots of this process and thus cannot represent the full dynamics of developmental processes. Second, considerable clutch variation exists within a given “wild-type” stock, let alone between “wild-type” stocks under use around the world. We have attempted to normalize such differences to some extent by noting the standard length of each specimen and also by comparing morphological features of specimens to standardized staging series [34,35]. In addition, we tried to select specimens that were representative of the clutch at each timepoint. Finally, imaging always is subject to limits of detection, so we try to avoid making declarative statements that a given skeletal element “appears” at a given time. That said, we have refined our methods to detect skeletal elements with the most sensitive techniques available. Our confocal imaging illustrates specifically skeletogenic cells prior to their secretion of abundant extracellular matrix. Also, we incubate and image our specimens with the vital dye Alizarin red while they are alive. As such, we avoid the severe reduction in Alizarin red binding that occurs after even brief periods in fixative and artificial buffers, for these chemicals leech mineral from skeletal tissues in the specimen. In summary, the FishFace Atlas addresses potential pitfalls of a variable developmental process and limitations of skeletal detection to provide the user an understanding of development of the pharyngeal arches and its skeleton in the embryonic and larval zebrafish.

## Availability and requirements

The data sets supporting the results of this article are included within the article (and its Additional files 1 and 2) or are available in the FishFace Atlas (https://www.facebase.org/fishface/home). There are no restrictions on use by non-academics. All users are free to download images or movies (please see Additional file 1).

## Abbreviations

Anatomical: Terminology and abbreviations used in the FishFace Atlas are based largely upon Cubbage and Mabee (1996) and Zebrafish Information Network ontology (http://zfin.org).

## Competing interests

The authors declare that they have no competing interests.

## Authors’ contributions

AD and CBK conceived of the study. MM and JD raised the fish. BFE, AD, BU, TRH, JTN, and MMS performed the imaging. BFE and CBK wrote the manuscript. All authors read and approved the final manuscript.

## Supplementary Material

Additional file 1 Relationship between the FishFace Atlas and FaceBase hub, Mechanism for adding material to FishFace, Instructions for downloading FishFace data. Click here for file

Additional file 2 ***Isolation and genomic cloning of a chondrocyte***-***specific transgenic line***, **sox9a**^**zc81Tg**^, ***from *Tg(foxp2**.**A**:**EGFP)zc42.** We have reported previously *sp7*:*EGFP* (formally called *Tg*(*sp7*:*EGFP*)*b1212*; [28]), a zebrafish line that illuminates osteoblast formation and distribution during bone development, but we needed its counterpart in cartilage development. Here, we characterize a novel transgenic insertion that has chondrocyte-specific expression. *Tg*(*foxp2*.*A*:*EGFP*)*zc42* was reported to have two bright domains of expression: 1) brain and 2) pharyngeal arches (Additional file 2: Figure S1A; [27]). The brain expression was expected from the known *foxp2* expression patterns, but the pharyngeal arch expression was unexpected, as *foxp2* is not expressed in this tissue [27]. Fish were isolated with bright expression domains in either brain or pharyngeal arches (Additional file 2: Figure S1B, Figure S1C), suggesting that at least two insertions of the Gateway construct were responsible for the two initial transgene expression domains. For the following reasons, we hypothesized that expression of the *Tg*(*foxp2*.*A*:*EGFP*) construct in pharyngeal arches was due to position-dependent genomic effects, similar to an enhancer trap [36]. Only the brain, and not the pharyngeal arch, expression domain of *Tg*(*foxp2*.*A*:*EGFP*)*zc42* could be recapitulated by injecting the *Tg*(*foxp2*.*A*:*EGFP*) construct into fertilized eggs (data not shown). Moreover, there was only one initial founder of *Tg*(*foxp2*.*A*:*EGFP*)*zc42* with the pharyngeal arch expression (J. Bonkowsky, pers. comm.). To test further the enhancer-trap hypothesis, we used zebrafish with only the pharyngeal arch expression domain (Additional file 2: Figure S1C) to identify the genomic locus of the *Tg*(*foxp2*.*A*:*EGFP*) insertion (see Construction and content). Inverse PCR revealed that the insertion site is linked to the known chondrocyte differentiation gene *sox9a*, approximately 120 kb upstream of the *sox9a* transcriptional start site in a 400 kb stretch of the genome devoid of known genes (Additional file 2: Figure S1D; see also Construction and content). Indeed, analyses of later-staged zebrafish larvae demonstrated that the pharyngeal arch expression was specific to developing chondrocytes (Figures 2, 3 and 4). Interestingly, the transgene inserted very close to an approximately 300 bp non-coding sequence that is conserved among medaka, stickleback, fugu, frog, mouse, and human (http://genome.ucsc.edu/cgi-bin/hgTracks?db=danRer7&position=Chr12:2153000-2307118). Due to its genetic linkage, the approved formal name of this transgenic line is *sox9a*^*zc81Tg*^ (http://www.zfin.org), which we will refer to hereafter as *sox9a*:*EGFP*. Click here for file
