# Supplementary material for: FishFace: interactive atlas of zebrafish craniofacial development at cellular resolution
Source: BMC Dev Biol. 2013 May 28;13:23. doi: 10.1186/1471-213X-13-23 (PMC3698193; doi:10.1186/1471-213X-13-23)
Supplement: Additional file 1 — Relationship between the FishFace Atlas and FaceBase hub, Mechanism for adding material to FishFace, Instructions for downloading FishFace data. [file 1471-213X-13-23-S1.docx]

**Appendix**

***Relationship between the FishFace Atlas and FaceBase hub***

FaceBase is an NIH U01-funded consortium of 11 projects focussing on human, mouse, and zebrafish craniofacial research. The FaceBase hub coordinates findings from these projects on a publicly-accessible, online website ([www.facebase.org](http://www.facebase.org)). Even though the FishFace Atlas is not one of the 11 FaceBase projects, it is hosted by the FaceBase hub.

***Mechanism for adding material to FishFace***

We envisage that the database can be expanded under the tutelage of the FaceBase hub with contributions from colleagues around the world working on imaging zebrafish craniofacial development and anatomy. The corresponding authors of this paper have agreed to advise FaceBase on the inclusion of contributor’s materials. Future additions to FishFace should be directed to the Kimmel lab (as indicated on the FishFace home page: <https://www.facebase.org/fishface/home>) or through the FaceBase help desk at [help@facebase.org](mailto:help@facebase.org).

***Instructions for downloading FishFace data***

***A) images***

Images can be downloaded directly from the FishFace Atlas by right-clicking on the image of interest. If the user would like higher-resolution images, then please contact the Kimmel lab.

***B) movies (and converting them to image stacks)***

(Note: these instructions are current for downloading and converting movies at the time of publication. We cannot guarantee that third-party video conversion resources will be available at these web addresses in the future.)

1) Using Firefox 20.0, download the Flash Video Downloader add-on (under tools/add-ons, also available at http://flashvideodownloader.org) and follow the instructions for installation.

2) Upon restarting Firefox, a grey arrow icon will appear on the toolbar line on the right side of webpage address line.

3) Go to FishFace and begin playing a movie. The arrow should change from grey to blue. Click on the arrow to download the movie as an .flv file format.

4) Open the .flv file in ImageJ version 1.46 (use the 32-bit version of ImageJ) using File/open. In the QT Movie Opener window, choose “Virtual Stack”.

5) Use File/save as/Image Stack to export a stack of single image files for viewing and analysis in ImageJ.

6) If you have difficulty opening the .flv file directly in ImageJ, go to the website http://www.zamzar.com/

7) Follow the instructions to convert .flv files to .mov formats. The converted file will be emailed to you.

8) You should be able to view the movie in Quicktime. If you have Quicktime Pro, you can export “movie to image sequence” which will generate an image stack for viewing in ImageJ.
