# Supplementary material for: FishFace: interactive atlas of zebrafish craniofacial development at cellular resolution
Source: BMC Dev Biol. 2013 May 28;13:23. doi: 10.1186/1471-213X-13-23 (PMC3698193; doi:10.1186/1471-213X-13-23)
Supplement: Additional file 2 — Isolation and genomic cloning of a chondrocyte-specific transgenic line, sox9azc81Tg, from Tg(foxp2.A:EGFP)zc42. We have reported previously sp7:EGFP (formally called Tg(sp7:EGFP)b1212; [28]), a zebrafish line that illuminates osteoblast formation and distribution during bone development, but we needed its counterpart in cartilage development. Here, we characterize a novel transgenic insertion that has chondrocyte-specific expression. Tg(foxp2.A:EGFP)zc42 was reported to have two bright domains of expression: 1) brain and 2) pharyngeal arches (Additional file 2: Figure S1A; [27]). The brain expression was expected from the known foxp2 expression patterns, but the pharyngeal arch expression was unexpected, as foxp2 is not expressed in this tissue [27]. Fish were isolated with bright expression domains in either brain or pharyngeal arches (Additional file 2: Figure S1B, Figure S1C), suggesting that at least two insertions of the Gateway construct were responsible for the two initial transgene expression domains. For the following reasons, we hypothesized that expression of the Tg(foxp2.A:EGFP) construct in pharyngeal arches was due to position-dependent genomic effects, similar to an enhancer trap [36]. Only the brain, and not the pharyngeal arch, expression domain of Tg(foxp2.A:EGFP)zc42 could be recapitulated by injecting the Tg(foxp2.A:EGFP) construct into fertilized eggs (data not shown). Moreover, there was only one initial founder of Tg(foxp2.A:EGFP)zc42 with the pharyngeal arch expression (J. Bonkowsky, pers. comm.). To test further the enhancer-trap hypothesis, we used zebrafish with only the pharyngeal arch expression domain (Additional file 2: Figure S1C) to identify the genomic locus of the Tg(foxp2.A:EGFP) insertion (see Construction and content). Inverse PCR revealed that the insertion site is linked to the known chondrocyte differentiation gene sox9a, approximately 120 kb upstream of the sox9a transcriptional start site in a 400 kb stretch of the genome [file 1471-213X-13-23-S2.pdf]

Eames et al.
Supplemental Figure 1

Isolation and genomic mapping of *sox9a:EGFP*, a novel transgenic insertion.

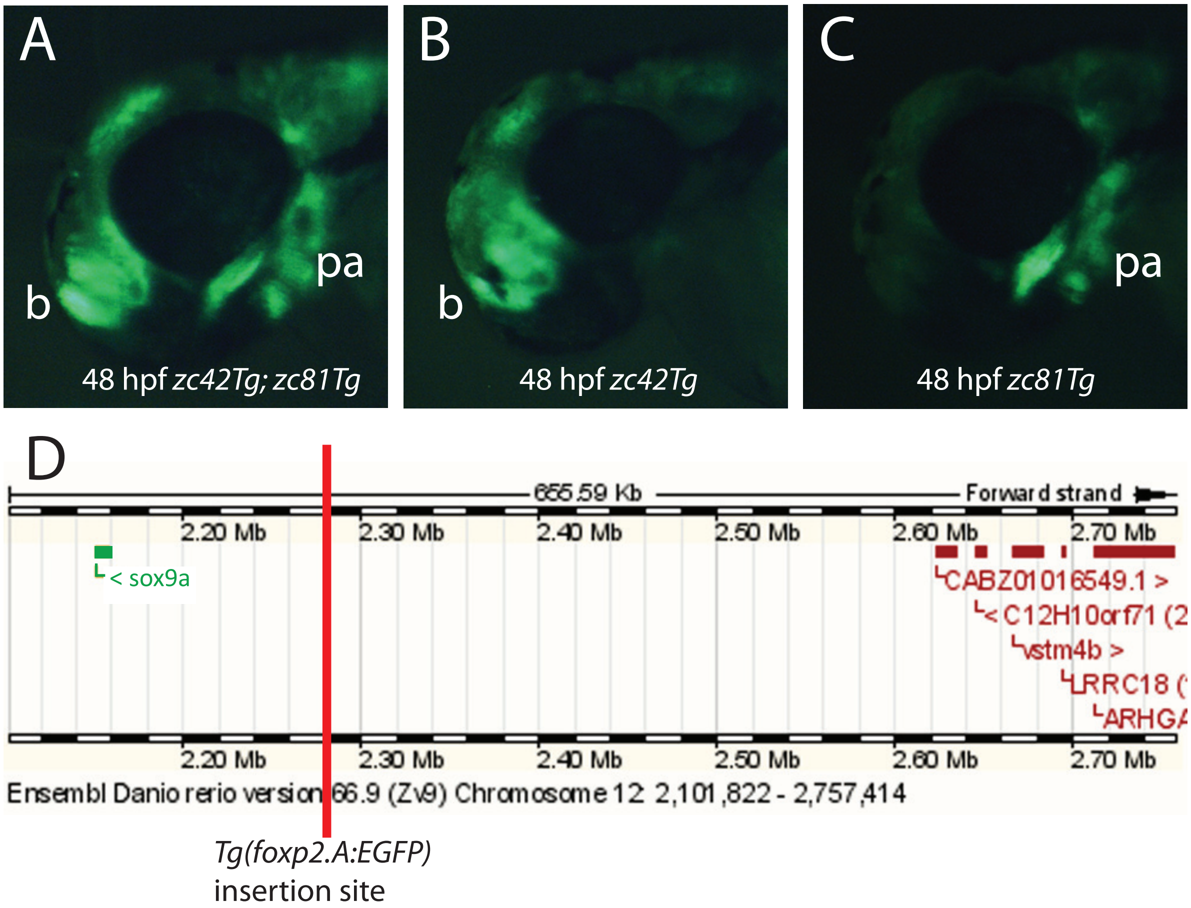

Fluorescent images (A-C) of lateral views of 48 hours post fertilization *Tg(foxp2.A:EGFP)zc42* embryos demonstrate the isolation of a novel transgenic insertion, resulting in the *sox9a:EGFP* expression domain. The initial stable line of *Tg(foxp2.A:EGFP)zc42* had two expression domains: brain and pharyngeal arches (A). These two domains independently segregated in offspring (B,C). Using inverse PCR, the genomic location of the insertion site of the pharyngeal arch expression domain was identified as being approximately 120kb upstream of the *sox9a* transcriptional start site in a 400kb stretch of the genome devoid of known genes (D). The official name of this transgenic line is $sox9a^{zc81Tg}$, which we refer to as *zc81Tg* in the atlas, and for clarity in this paper *sox9a:EGFP* Abbreviations: b=brain; pa=pharyngeal arches.
